# Effects on *Pseudomonas plecoglossicida* 2,4-D and Humic Substances on the Growth, Pigment Indices and Concentration of Hormones in Wheat Seedlings Grown under Water Deficit

**DOI:** 10.3390/microorganisms11030549

**Published:** 2023-02-21

**Authors:** Arina Feoktistova, Maxim Timergalin, Sergey Chetverikov, Aleksey Nazarov, Guzel Kudoyarova

**Affiliations:** 1 Ufa Institute of Biology, Ufa Federal Research Centre, RAS, Prospekt Oktyabrya 69, Ufa 450054, Russia; 2 Department of Environment and Rational Use of Natural Resources, Faculty of Business Ecosystem and Creative Technologies, Ufa State Petroleum Technological University, ul. Kosmonavtov 1, Ufa 450064, Russia

**Keywords:** *Pseudomonas plecoglossicida* 2,4-D, humic substances, water deficit, abscisic acid, cytokinins, NBI, chlorophyll, flavonoids

## Abstract

The search for ways to increase plant productivity in drought conditions is of fundamental importance, since soil moisture deficiency is widespread and leads to critical crop losses. The aim of this study was to identify the effects of plant growth-promoting bacteria and humic substances on the growth, chlorophyll, flavonoids, nitrogen balance index, and concentration of cytokinins and abscisic acids in wheat plants grown in the laboratory under conditions of water deficit. An increase in the accumulation of plant mass was shown during the treatment of wheat plants with *Pseudomonas plecoglossicida* 2,4-D and humic substances in these conditions. It has been shown that stimulating plant growth is associated with increased root growth, which leads to an increase in the nitrogen balance index, chlorophyll, and flavonoid concentrations in treated plants. The detected increase in the concentration of chlorophyll in plants treated with *P. plecoglossicida* 2,4-D correlated with a decrease in the concentration of abscisic acid in plant shoots and, in plants treated with humates, with an increase in the concentration of cytokinins in shoots. The higher efficiency of treating plants with a combination of bacteria and humic substances than with any of them individually may be associated with the additive effect of these treatments on the hormonal balance.

## 1. Introduction

Plant growth-promoting (PGP) bacteria became very popular due to their ability to stimulate plant growth [1,2,3]. This effect of bacteria on plant growth is explained by the improvement under their influence of mineral nutrition due to the solubilization of phosphates [4,5] and potassium [6], as well as by the ability to assimilate atmospheric nitrogen, which is characteristic not only of symbiotic but also of many free-living (nonsymbiotic) bacteria [7]. The production of hormones by bacteria (such as auxins, cytokinins, and gibberellins) that stimulate plant growth due to their direct influence on the processes of cell division and elongation is also widely discussed [3,8]. This action of bacteria promotes an increase in plant productivity, and therefore, it is not surprising that preparations based on such bacteria are increasingly used in agriculture [9]. An increasing world population requires more food production, while the use of plant growth-stimulating microbes (PGPM) is an attractive method to increase crop yields.

The growth-promoting capacity of bacteria is manifested not only in favorable environments but even more frequently in stressful conditions [10,11,12]. Many reports have shown an increase in drought tolerance in terms of supporting plant growth under the influence of PGP bacteria [13]. The ability of bacteria to protect plants from drought is associated with plant protection from the consequences of oxidative stress accompanying drought, which causes, in particular, the breakdown of pigments necessary for photosynthesis [14]. Finding ways to increase plant productivity in drought conditions is of fundamental importance, since the shortage of soil moisture is widespread and leads to critical crop losses [15]. 

Alongside with the use of PGP bacteria, the promotion of plant growth by humic substances (HSs, products of degradation of organic matter extracted from brown coal, peat, and other sources [16,17,18]) is well documented in the literature. Data on the effects of humic acids on plant growth and productivity are less abundant than those about PGP bacteria but still frequently reported [16]. Humic substances are assumed to produce both direct and indirect effects on plant growth. The indirect effect of humic acids is associated with soil structure modification under their influence in the rhizosphere region and with an increase in the availability of mineral nutrients for plants, while their direct effect is manifested in changes in plant metabolism and development [19,20]. The direct effect of humic acids on the growth and development of plants is a manifestation of their hormone-like activity [21,22,23]. Auxins (mainly in the form of indolylacetic acid) [20,24] and cytokinins (in the form of isopentenyladenine) were detected in humic acid preparations [25]. Humic acids affect enzyme activity, gene expression, and proton pump activity in the same way as the plant hormone auxin. Some publications indicate that humic substances have auxin-like activity, confirmed by their influences on the architecture and metabolism of the roots [26]. The hormone-like activity of humic preparations is one of the advantages of organic fertilizers over chemical fertilizers. 

HSs have been successfully used for increasing plant productivity in drought conditions [27]. Furthermore, a recent review recommends the combination of PGP bacteria with HSs as an alternative for sustainable agriculture [28]. However, it is emphasized in another review [28] that the number of reports describing the action of PGP bacteria in combination with HSs is extremely low in comparison with the great potential of this treatment option. We managed to find only one article showing a positive effect of the combination of PGP bacteria with HSs on plant growth in drought conditions [29]. 

The ability of PGP bacteria to synthesize plant hormones and influence the concentration of phytohormones in plants is considered one of the main mechanisms that stimulate plant growth [3,30,31]. We have recently shown that the combination of PGP bacteria with HSs increases the concentration of plant hormones auxins in the roots thereby stimulating root branching [32]. However, the effects of this treatment option on the concentration of other hormones have not been studied. Meanwhile, cytokinins and abscisic acid are plant hormones that, more often than auxins, are involved in plant responses to drought [33,34]. There are many indications that exogenous cytokinins influence stress resistance. Thus, spraying the leaves of creeping bent grass (*Agrostis stolonifera*) with a solution of cytokinin reduced the stress-induced decrease in the content of chlorophyll and activity of photosynthesis [35]. Transgenic plants with increased production of cytokinins were successfully employed for improvement of drought resistance in rice [36]. Considering the above, the aim of this study was to identify the effects of PGP and HSs bacteria on the growth and concentration of cytokinins and abscisic acids in wheat plants grown under conditions of water deficit (WD).

## 2. Materials and Methods

The objects of research were plants of spring bread wheat, *Triticum aestivum* L. cv. Kinelskaya, an auxin-producing bacterial strain of *Pseudomonas plecoglossicida* 2,4-D (2,4-D) from the collection of the Ufa Institute of Biology UFIC RAS (Russia), and humic substances (HSs) obtained from brown coal. *Pseudomonas plecoglossicida* 2,4-D received its name due to its resistance to the herbicide 2,4-D.

### 2.1. Bacterial Strain

*Pseudomonas plecoglossicida* 2,4-D bacteria were cultivated for 4 days in Erlenmeyer flasks on a thermostatically controlled shaker (160 rotations per minute) at 28 °C in the King B nutrient medium (g L^−1^): peptone, 20.0; glycerol, 10.0; K_2_HPO_4_, 1.5; and MgSO_4_·7H_2_O, 1. 

### 2.2. Experiments with Plants

Seedlings were grown during a 14-h photoperiod, day/night temperature regimes of 26/22 °C, and irradiance of 400 µmol m^−2^ s^−1^ from mercury-arc and sodium vapor lamps Osram Fluora fluorescent (Germany, Munich). Wheat seeds were previously sterilized and germinated. 3-day-old seedlings were transplanted into plastic vessels with sand. Sand was used because of the absence of humic substances in it. Prior to experiments, sand was sterilized by calcinations to exclude the presence of undesirable bacteria. In the control variant of the experiment (normal conditions), plants were watered daily to maintain humidity levels at 50% and 60% of the total moisture capacity of sand before and after watering, respectively. Water scarcity was modeled by maintaining humidity at 20–30% of the total moisture capacity of sand before and after watering, respectively.

Inoculation of plants with 1 mL (per plant) of bacteria suspension (10^8^ CFU mL^−1^) or treatment with 1 mL (per plant) of humates (0.1% aqueous solution) was carried out 3 days after planting by spraying leaves and ground.

Growth indicators (shoot and root mass and shoot length) were evaluated 2 weeks after exposure.

### 2.3. Determination of Chlorophyll, Flavonoids and Nitrogen Balance Index

The chlorophyll, flavonoids content, and nitrogen balance index (NBI) were measured by a portable plant analyzer, Dualex Scientific+ (Force-A, Paris, France), on the 7th day after plant treatment.

### 2.4. Extraction of Abscisic Acid

The hormone content was determined by enzyme immunoassay 3 days after treatment. To do this, the shoots and roots were homogenized and extracted with 80% ethyl alcohol. The alcohol extract was evaporated to an aqueous residue; after centrifugation of the latter, aliquots of the supernatant were selected for further purification. Purification and concentration of ABA were carried out according to a modified scheme with a decrease in volume [5]. After adjusting the pH to 2.5 with HCl the extract was partitioned three times with diethyl (the ratio of organic to aqueous phases used was 3:1). The combined extract is the primary ether extract. Subsequently, ABA was transferred from the organic phase into an equal amount of 1% sodium hydrocarbonate (pH 7–8) (the ratio of the aqueous to organic phases was 1:3) (sodium hydrocarbonate extract). Readjusting the pH of the aqueous phase to 2.5 and re-extracting with ether gave the secondary ether extract. Reducing the amount of extractant at each stage of extraction and re-extraction increased the selectivity of hormone recovery, which was not less than 80%. 

### 2.5. Purificaiton of Cytokinins

Cytokinins from the aqueous residue were concentrated on a pre-wetted C18 column (Waters, Milford, MA, USA), and, after washing, the column was loaded with 20 mL of distilled water and then eluted with 5 mL of 80% ethanol. After solvent evaporation, the dry residue was dissolved in 0.02 mL of 80% ethanol and applied to TLC plants for chromatography [37,38]. TLC was carried out on pre-coated Merck silica gel 60 F-254 plates, which were developed in 2-butanol:14 M NH_4_ OH:H_2_O (6:1:2 *v*/*v*, upper phase). Cytokinin zones were eluted with 0.1 M phosphate buffer at pH 7.4 for 12 h, and elutants were added directly to microplate wells in several dilutions for immunoassay. Recovery of cytokinins was not less than 80%.

### 2.6. Enzyme Immunoassay

Hormones were immunoassayed using the corresponding specific antibodies. Enzyme Linked Immunosorbent Assay (ELISA) was carried out using competitive protocol as described [5,37,38]. On the first step of the procedure, protein-phytohormone conjugate was passively adsorbed to a 96-well polystyrene microtiter plate in phosphate buffer (pH 7.5) at 37 °C for 1.5 h. The plate was washed three times with phosphate-buffered saline containing 0.05% Tween 20 and 0.5% ovalbumin (pH 7.2). A mixture of 10 µL of different concentrations of hormone standard or sample plus 180 µL of antisera was added to each well and incubated for 1 h at 37 °C. On this step, the sample antigen competes with a reference antigen (protein-phytohormone conjugate bound to the well walls) for binding to a specific amount of an antibody. Unbound rabbit serum was washed away, and goat antirabbit IgG conjugated to peroxidase was incubated with the adsorbed antigen-antibody complex for 1 h at 37 °C. All wells were again washed, and the substrate solution, consisting of o-phenylene-diamine in 0.3 M phosphate buffer pH 5.5:3% hydrogen peroxide in the ratio of 10 mL:15 mL:50 µL, was added. The color developed was quantitated at 492 nm with a microphotometer (Uniplan, Moscow, Russia). The reliability of the method was confirmed by comparison of its results with the data obtained with HPLC combined with mass spectrometry [38].

### 2.7. Statistical Data Processing

Data are expressed as means ± S.E., which were calculated in all treatments using MS Excel. Significant differences between means were analyzed by one-way analysis of variance and Duncan’s test to discriminate means. The data were processed using Statistica version 10 software (Statsoft, Moscow, Russia).

## 3. Results

Analysis of variance showed that there were differences in shoot length and mass, root mass, chlorophyll and flavonoid contents, nitrogen balance index (NBI), content of abscisic acid, and cytokinins in shoots and roots between groups of plants indicated in the Table 1.

### 3.1. Growth Characteristics

**Figure 1:**
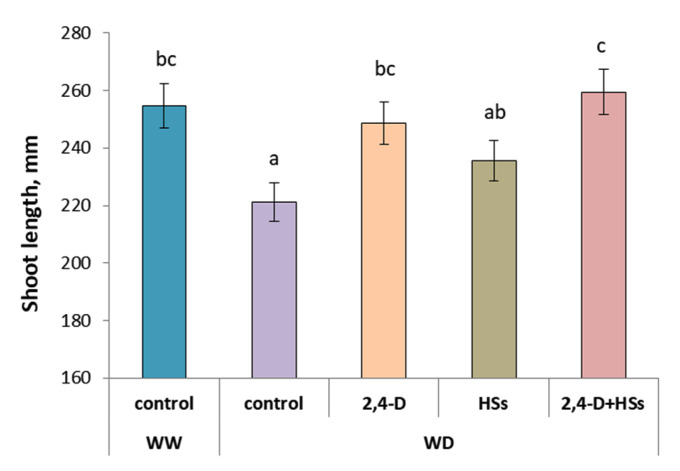
Shoot length of wheat plants 14 days after treatment with *Pseudomonas plecoglossicida* 2,4-D (2,4-D), humic substances (HSs) and their combinations (2,4-D + HSs) under well-watering (WW) and water deficit (WD) conditions. Statistically different means are marked with different letters, *p* ≤ 0.05, n = 15 (ANOVA followed by Duncan’s test).

**Figure 2:**
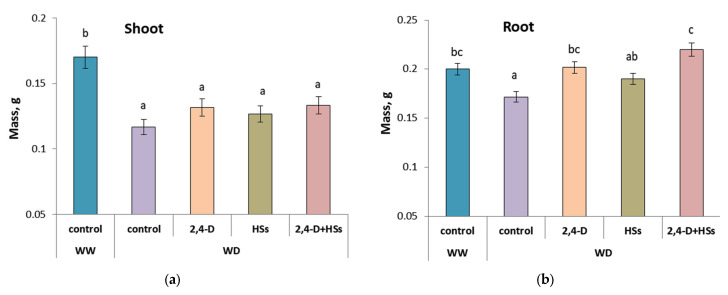
Mass of shoots (**a**) and roots (**b**) of wheat plants 14 days after treatment with *Pseudomonas plecoglossicida* 2,4-D (2,4-D), humic substances (HSs) and their combinations (2,4-D + HSs) under well-watering (WW) and in water deficit (WD) conditions. Statistically different means are marked with different letters, *p* ≤ 0.05, n = 15 (ANOVA followed by Duncan’s test).

In the absence of bacterial and HSs treatments, a water deficit significantly inhibited the growth of wheat plants, which was manifested in the shortening of shoots (Figure 1, Table 2) and lower mass of both shoots and roots compared with well-watered control plants (Figure 2a,b; Table 3 and Table 4). Either bacterial inoculation or HS treatment applied individually or in combination accelerated shoot elongation so that their shoot length did not differ from that of well-watered control plants (Figure 1, Table 2). There was no difference in the shoot length between the plants treated with either *P. plecoglossicida* 2,4-D or humic substances applied individually. However, the combination of bacteria and HSs was more effective, resulting in longer shoots than in plants treated with HSs alone.

The mass of shoots of plants that experienced water deficits was lower than that of well-watered plants, and the increase in the mass of shoots for all treatment options was statistically insignificant (Figure 2a, Table 3). The WD-induced reduction in root mass was less than the reduction in shoot mass. As a result the root-to-shoot mass ratio increased from 1.2 in the control well-watered plants to about 1.5-1.7 in WD-plants.

The root mass of control plants (untreated with either bacteria or HS) grown under a water deficit was significantly lower than in the well-watered control (Figure 2b, Table 4). Bacterial inoculation, applied individually and in combination with HSs treatment, significantly increased root mass. In plants treated with HSs alone, the root mass did not differ from either WD control plants or plants treated with bacteria alone, but was significantly lighter than in plants treated with a combination of bacteria and HSs. Thus, the combined application of bacteria and HSs proved to be more effective, resulting in significantly larger root masses, compared with the WD control or HSs-treated plants.

### 3.2. Pigments and Nitrogene Balance Index

**Figure 3:**
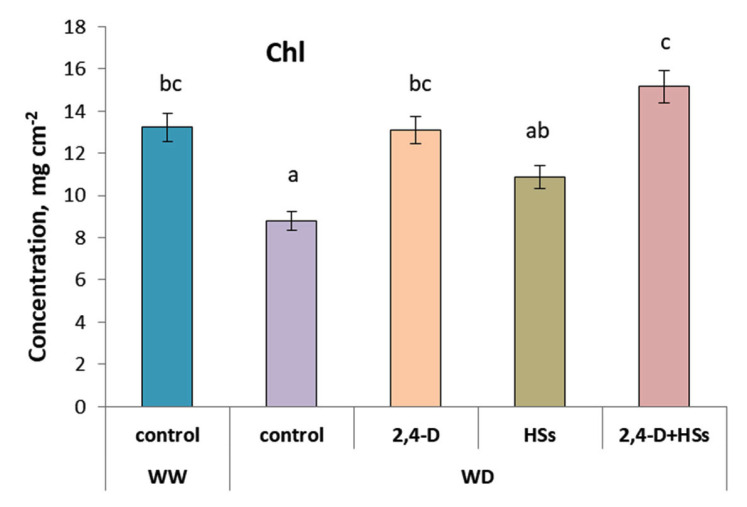
Concentration of chlorophyll (Chl) in leaves of wheat plants 7 days after treatment with *Pseudomonas plecoglossicida* 2,4-D (2,4-D), humic substances (HSs) and their combinations (2,4-D + HSs) under well-watering (WW) and in water deficit (WD) conditions. Statistically different means are marked with different letters, *p* ≤ 0.05, n = 30 (ANOVA followed by Duncan’s test).

Chlorophyll concentration was decreased by the water deficit (Figure 3; Table 5), while all the treatments of WD-plants increased concentration of chlorophyll up to the level of control well-watered plants. There was no significant difference in chlorophyll concentration between the plants treated with either bacteria or HS applied alone. However, their combination proved to be more effective, resulting in a significantly higher concentration of chlorophyll compared with the use of HSs alone.

**Figure 4:**
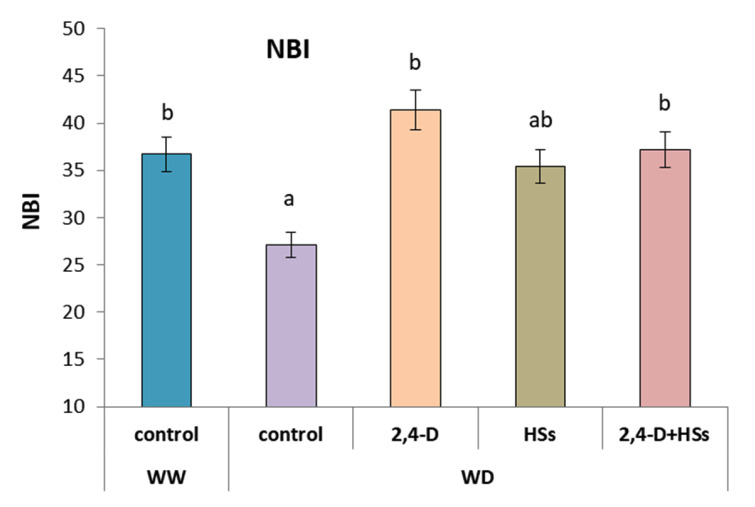
Nitrogen balance index (NBI) in leaves of wheat plants 7 days after treatment with *Pseudomonas plecoglossicida* 2,4-D (2,4-D), humic substances (HSs) and their combinations (2,4-D + HSs) under well-watering (WW) and in water deficit (WD) conditions. Statistically different means are marked with different letters, *p* ≤ 0.05, n = 30 (ANOVA followed by Duncan’s test).

In plants untreated with either HSs or bacteria, NBI in leaves was decreased by WD, while each treatment and their combination increased this indicator up to the level of well-watered control plants (Figure 4, Table 6). Furthermore, NBI in plants treated with HSs alone did not differ significantly from control WD plants.

**Figure 5:**
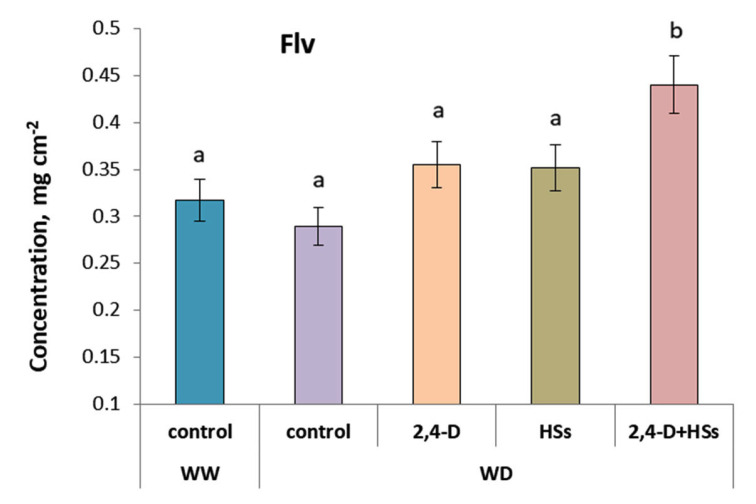
Concentration of flavonoids (Fvl) in leaves of wheat plants 7 days after treatment with *Pseudomonas plecoglossicida* 2,4-D (2,4-D), humic substances (HSs) and their combinations (2,4-D + HSs) under well-watering (WW) and in water deficit (WD) conditions. Statistically different means are marked with different letters, *p* ≤ 0.05, n = 30 (ANOVA followed by Duncan’s test).

Unlike chlorophyll concentration, that of flavonoids was not significantly decreased by WD but was increased by a combination of humic substances and bacteria (Figure 5, Table 7).

### 3.3. Concentration of Hormones

**Figure 6:**
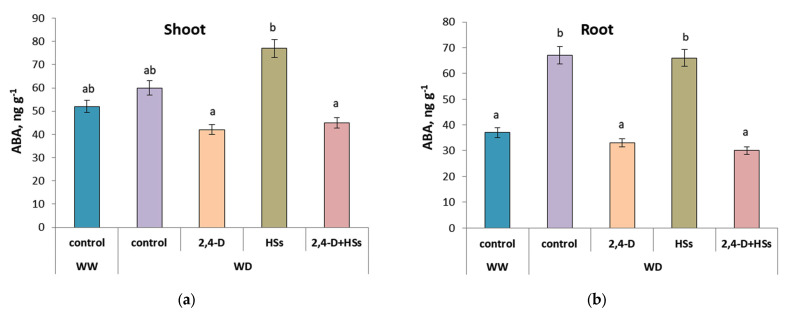
ABA concentration in shoots (a) and roots (b) of wheat plants 3 day after treatment with *Pseudomonas plecoglossicida* 2,4-D (2,4-D), humic substances (HSs) and their combinations (2,4-D + HSs) under well-watering (WW) and in water deficit (WD) conditions. Statistically different means are marked with different letters, *p* ≤ 0.05, n = 9 (ANOVA followed by Duncan’s test).

The tendency of the WD-induced ABA accumulation in the shoots of the control plants compared with well-watered plants was statistically insignificant (Figure 6a, Table 8). The ABA concentration in the shoots of plants treated with HSs was significantly higher than in plants treated with bacteria (alone or in combination with HSs).

Water deficit increased the ABA concentration in the roots of control plants (untreated with either bacteria or HSs) as well as in the roots of plants treated with humic substances (Figure 6b, Table 9). Bacterial treatment (either alone or in combination with HSs) prevented WD-induced ABA accumulation.

**Figure 7:**
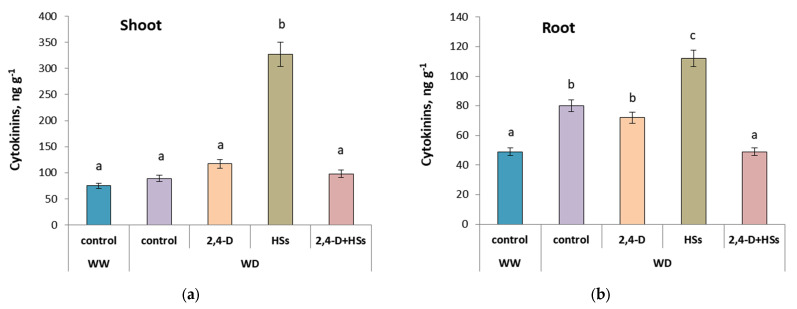
Cytokinins concentration in shoots (**a**) and roots (**b**) of wheat plants 3 day after treatment with *Pseudomonas plecoglossicida* 2,4-D (2,4-D), humic substances (HSs) and their combinations (2,4-D + HSs) under well-watering (WW) and in water deficit (WD) conditions. Statistically different means are marked with different letters, *p* ≤ 0.05, n = 9 (ANOVA followed by Duncan’s test).

The cytokinin concentration in the shoots was significantly increased only by HS treatment (Figure 7a, Table 10). All other options (including control plants under WD conditions) did not significantly influence cytokinin concentrations in the shoots. 

WD increased the concentration of cytokinins in the roots of all plants (Figure 7b, Table 11), except for those treated with a combination of humic substances and bacteria. The concentration of cytokinins was highest in the roots of plants treated with HSs applied alone.

Generalization of the obtained results shows that both bacterial treatment and HS treatment reduced the harmful effects of WD on wheat plants. Shoot length and root mass decreased under the action of WD in the control plants (not treated with either bacteria or HSs), but increased in plants treated with HSs and bacteria up to the level in well-watered plants. The nitrogen balance index (NBI) in the leaves of control plants, also reduced by WD, reached the level of the well-watered control in plants treated with either bacteria or HSs, applied alone or in combination with each other. In a number of aspects, the combined treatment of the plants with bacteria and HSs proved to be more effective. Thus, under WD conditions, a statistically significant increase in shoot length compared with control plants was found only in the case of treatment with a combination of HS and bacteria, while the shoot length of plants treated with either bacteria or HS alone was intermediate between the control and combined treatments. The same was shown for flavonoid content, which increased only in plants treated with the combination of bacteria and HS and not when both were used individually.

## 4. Discussion

The water deficit (WD) adversely affected plant growth. WD reduced the root mass of control plants, while it increased it to the level of well-watered plants after treatment with bacteria and HS. Thus, the treatments mitigated the detrimental effect of WD on the root mass. Root response treatments appeared to increase their ion uptake capacity, as evidenced by an increase in the nitrogen balance index (NBI) in treated plants. 

The enhancement of root growth caused by *P. plecoglossicida* 2,4-D can be associated with the ability of this bacterial strain to produce auxins and increase their concentration in the roots, thereby increasing the root mass [32]. This explanation sounds reasonable, since auxins are known to stimulate the growth of lateral roots [39]. Humic substances also increased the concentration of auxins in the roots [18]. The positive effect on root mass was greatest with the combined treatment of plants with bacteria and HS and the smallest when plants were treated with HS alone. Less accumulation of root mass may be due to the high concentration of cytokinins in the roots of HS-treated plants, while these hormones are known to inhibit root growth [40,41]. The additive effect of bacteria and HSs on root growth may be associated with the prevention by bacteria of HS-induced cytokinin accumulation. Since auxins are known to activate cytokinin oxidase [42], auxins produced by bacteria can activate oxidative destruction of cytokinin excess, thus compensating for the negative effect of HSs on root growth. 

Declining chlorophyll content in untreated plants under WD is another indicator of detrimental stress effects. This is considered a typical symptom of oxidative stress and stress-induced inhibition of photosynthesis [43]. In the present experiments, the treatment of wheat plants with bacteria and HSs maintained higher chlorophyll content under WD conditions and increased its concentration up to the level of the well-watered plants. The increase in chlorophyll concentration was most pronounced in the plants treated with bacteria alone and in their combination with humates. The importance of this effect is confirmed by its relationship with changes in plant biomass. A high correlation between chlorophyll concentration and plant mass was detected (r=0.85). 

Unlike chlorophyll, the concentration of flavonoids was not affected by drought in the present experiments. Flavonoids perform many functions in plants, including plant development through their participation in cell wall synthesis, symbiotic interaction with mycorrhizal fungi and rhizobia, and defense against fungal pathogens [44]. Their ability to interact with a wide range of other molecules, including reactive oxygen species (ROS), underlies their antioxidant activity, protecting organisms from oxidative damage [45]. Given these characteristics of flavonoids, it is not surprising that their highest concentration in plants treated with a combination of bacteria and HSs was associated with the highest concentration of chlorophyll in plants as an indicator of reduced oxidative damage.

Interestingly, effects of bacterial treatments in our experiments were associated with a decrease in the concentration of ABA in the plant. It is known that ABA is involved in the accelerated degradation of chlorophyll [46]; therefore, a decrease in the concentration of this hormone may contribute to an increase in chlorophyll concentration. A reduced concentration of ABA was found both in the shoots and in the roots of plants treated with bacteria, either separately or in combination with HS. It is known that some bacteria catabolize ABA, thereby reducing the concentration of this hormone in the inoculated plants [47]. The decrease in ABA concentration detected in plants inoculated with *P. plecoglossicida* 2,4-D in the present experiments indicates the ability of this bacterial strain to catabolize ABA. It would be interesting to test this hypothesis in the future.

The concentration of ABA in the leaves of plants treated with humates did not decrease in comparison with the control WD-plants, while the content of chlorophyll increased upon the treatment. This was likely to be due to the effect of HS on cytokinin concentration. Cytokinin-like activity was found in various humic substances [25], and the treatment of wheat plants with HSs increased cytokinin concentration in the leaves [18]. It is known that cytokinins delay senescence and the breakdown of chlorophyll in detached leaves [48]. These homones act as ABA antagonists, preventing chlorophyll degradation [49]. This explains the high concentration of chlorophyll in the leaves of plants treated with HSs, with increased the concentration of cytokinins. This explains the high concentration of chlorophyll in the leaves of plants treated with HSs, along with the increased content of cytokinins. Nevertheless, chlorophyll concentration in HS-treated plants was lower than in plants treated with a combination of HSs with bacteria. This effect can be explained by the increased concentration of ABA found in the leaves of HS-treated plants.

## 5. Conclusions

Thus, the analysis of the obtained results shows that the inoculation of wheat plants with the *Pseudomonas plecoglossicida* 2,4-D strain and their treatment with humic substances mitigated the negative impact of water deficit on root growth while maintaining their ability to absorb ions, which manifests itself in an increase in NBI. The treatments also prevented WD-induced decline in the content of pigments involved in photosynthesis (which prevented the drought induced decline in their content in leaves), which led to an improvement of growth rate of the plants (and an increase in shoot length) under conditions of water deficit. The effect of treatments on the content of chlorophyll in the leaves of plants can be explained by the ability of this bacterial strain to reduce the concentration of abscisic acid in wheat plants, as well as an increase in the concentration of cytokinin in plants treated with HS. This explanation is based on the known antagonistic effects of cytokinins and ABA on chlorophyll concentration: accelerated chlorophyll destruction by ABA and its prevention by cytokinins.

The combination of HSs and bacteria proved to be more effective than each of the treatments applied separately. Thus, the concentration of flavonoids in the leaves was highest with the combined treatment of the wheat plants. Since flavonoids are known to act as antioxidants, their high concentration in the plants should help them tolerate the oxidative stress that accompanies drought. A greater effect from the combined treatment of wheat plants with HS and bacteria was also manifested in the maximum root mass revealed in this variant. The additive effect of bacteria and HS on root growth may be associated with the prevention by bacteria of HS-induced cytokinin accumulation. Since cytokinins are known to inhibit root growth and auxins produced by bacteria can activate the oxidative destruction of cytokinin excess, the lower concentration of cytokinins in the roots of the plants treated with a combination of HSs and bacteria than in those treated with HSs alone may lead to increased accumulation of root biomass.

Similar to the effects on root mass, combined treatment of plants with HSs and bacteria produced the greatest effect on the level of chlorophyll in plants, and the high correlation between chlorophyll content and accumulation of plant biomass highlights the importance of this parameter. The increased accumulation of chlorophyll in treated plants is probably due not only to the hormonal effect on chlorophyll metabolism but also to an increase in root mass, which improves the ability of roots to absorb nitrogen, which is necessary for the synthesis of chlorophyll.

Thus, the combination of bacteria with humic substances is a promising technique for increasing the drought resistance of cultivated plants. However, since the present experiments were carried out on wheat seedlings, further experiments with more mature plants are needed to prove the applicability of the combined treatment of plants with bacteria and humic substances in agricultural practice.

## Figures and Tables

**Table 1:** One-way ANOVA test of significance in some variables between groups: control plants untreated with either bacteria or HSs and plants grown under water deficit (control, plants treated with *Pseudomonas plecoglossicida* 2,4-D (2,4-D), humic substances (HSs) and their combinations (2,4-D + HSs). Values in the cells correspond to p for the difference between the mean values; *p* ≤ 0.05 is marked with *; *p* ≤ 0.01—with **.

Variables		Sum of Squares	df	Mean Square	F	Sig.
Shoot length	Between Groups	16,419.850	4	4104.963	4.405	0.003 **
	Within Groups	76,413.966	82	931.878		
	Total	92,833.816	86			
Shoot mass	Between Groups	0.008	4	0.002	5.243	0.003 **
	Within Groups	0.009	25	0.000		
	Total	0.017	29			
Root mass	Between Groups	0.008	4	0.002	3.218	0.029 *
	Within Groups	0.015	25	0.001		
	Total	0.022	29			
Chlorophyll	Between Groups	240.626	4	60.156	7.936	0.000 **
	Within Groups	341.097	45	7.580		
	Total	581.723	49			
Flavonoids	Between Groups	0.129	4	0.032	3.707	0.011 *
	Within Groups	0.393	45	0.009		
	Total	0.522	49			
NBI	Between Groups	1104.358	4	276.089	2.134	0.092
	Within Groups	5822.303	45	129.385		
	Total	6926.661	49			
ABA in Shoot	Between Groups	4778.578	4	1194.644	5.828	0.002 **
	Within Groups	4509.929	22	204.997		
	Total	9288.507	26			
ABA in Roots	Between Groups	8081.933	4	2020.483	8.204	0.000 **
	Within Groups	5910.957	24	246.290		
	Total	13,992.890	28			
Cytokinins in Soot	Between Groups	6449.054	4	1612.264	11.601	0.002 **
	Within Groups	1111.810	8	138.976		
	Total	7560.865	12			
Cytokinins in Roots	Between Groups	12,7381.298	4	31,845.325	4.380	0.031 *
	Within Groups	65,428.424	9	7269.825		
	Total	192,809.722	13			

**Table 2:** ANOVA test for shoot length followed by Duncan test. Values in the cells correspond to p for the difference between the mean values; *p* ≤ 0.05 is marked with *; *p* ≤ 0.01—with ** for control plants untreated with either bacteria or HSs and plants grown under water deficit (WD): control, plants treated with *Pseudomonas plecoglossicida* 2,4-D (2,4-D), humic substances (HSs) and their combinations (2,4-D + HSs).

Groups	Groups	{1}	{2}	{3}	{4}	{5}
	Mean Values	254.89	221.64	248.59	235.29	259.04
1	control		0.002448 **	0.543450	0.073258	0.689022
2	WD control	0.002448 **		0.012720 *	0.188109	0.000720 **
3	WD, 2,4-D	0.543450	0.012720 *		0.199237	0.345724
4	WD, HSs	0.073258	0.188109	0.199237		0.034495 *
5	WD, 2,4-D + HSs	0.689022	0.000720 **	0.345724	0.034495 *	

**Table 3:** ANOVA test for shoot mass followed by Duncan test. Values in the cells correspond to p for the difference between the mean values; *p* ≤ 0.05 is marked with *; *p* ≤ 0.01—with ** for control plants untreated with either bacteria or HSs and plants grown under water deficit (WD): control, plants treated with *Pseudomonas plecoglossicida* 2,4-D (2,4-D), humic substances (HSs) and their combinations (2,4-D + HSs).

Groups	Groups	{1}	{2}	{3}	{4}	{5}
	Mean Values	0.16500	0.11667	0.13167	0.12667	0.13333
1	control		0.000375 **	0.011116 *	0.004045 **	0.013839 *
2	WD control	0.000375 **		0.226659	0.390416	0.194972
3	WD, 2,4-D	0.011116 *	0.226659		0.666721	0.885799
4	WD, HSs	0.004045 **	0.390416	0.666721		0.590794
5	WD, 2,4-D + HSs	0.013839 *	0.194972	0.885799	0.590794	

**Table 4:** ANOVA test for root mass followed by Duncan test. Values in the cells correspond to p for the difference between the mean values; *p* ≤ 0.05 is marked with *; *p* ≤ 0.01—with ** for control plants untreated with either bacteria or HSs and plants grown under water deficit (WD): control, plants treated with *Pseudomonas plecoglossicida* 2,4-D (2,4-D), humic substances (HSs) and their combinations (2,4-D + HSs).

Groups	Groups	{1}	{2}	{3}	{4}	{5}
	Mean Values	0.20000	0.17167	0.20167	0.19000	0.22000
1	control		0.028929 *	0.888529	0.402758	0.118301
2	WD control	0.028929 *		0.024893 *	0.130208	0.000645 **
3	WD, 2,4-D	0.888529	0.024893 *		0.358522	0.130208
4	WD, HSs	0.402758	0.130208	0.358522		0.024893 *
5	WD, 2,4-D + HSs	0.118301	0.000645 **	0.130208	0.024893 *	

**Table 5:** ANOVA test for chlorophyll content followed by Duncan test. Values in the cells correspond to p for the difference between the mean values; *p* ≤ 0.05 is marked with *; *p* ≤ 0.01—with ** for control plants untreated with either bacteria or HSs and plants grown under water deficit (WD): control, plants treated with *Pseudomonas plecoglossicida* 2,4-D (2,4-D), humic substances (HSs) and their combinations (2,4-D + HSs).

Groups	Groups	{1}	{2}	{3}	{4}	{5}
	Mean Values	13.226	8.7745	13.101	10.867	15.148
1	control		0.008954 **	0.933932	0.165929	0.202602
2	WD control	0.008954 **		0.009923 **	0.166006	0.000207 **
3	WD, 2,4-D	0.933932	0.009923 **		0.178623	0.201437
4	WD, HSs	0.165929	0.166006	0.178623		0.012056 *
5	WD, 2,4-D + HSs	0.202602	0.000207 **	0.201437	0.012056 *	

**Table 6:** ANOVA test for NBI content followed by Duncan test. Values in the cells correspond to p for the difference between the mean values; *p* ≤ 0.05 is marked with *; *p* ≤ 0.01—with ** for control plants untreated with either bacteria or HSs and plants grown under water deficit (WD): control, plants treated with *Pseudomonas plecoglossicida* 2,4-D (2,4-D), humic substances (HSs) and their combinations (2,4-D + HSs).

Groups	Groups	{1}	{2}	{3}	{4}	{5}
	Mean Values	36.669	27.130	41.505	35.375	37.222
1	control		0.049428 *	0.328193	0.779825	0.904928
2	WD control	0.049428 *		0.005993 **	0.079307	0.048755 *
3	WD, 2,4-D	0.328193	0.005993 **		0.232100	0.356516
4	WD, HSs	0.779825	0.079307	0.232100		0.708877
5	WD, 2,4-D + HSs	0.904928	0.048755 *	0.356516	0.708877	

**Table 7:** ANOVA test for flavonoids content followed by Duncan test. Values in the cells correspond to p for the difference between the mean values; *p* ≤ 0.05 is marked with *; *p* ≤ 0.01—with ** for control plants untreated with either bacteria or HSs and plants grown under water deficit (WD: control, plants treated with *Pseudomonas plecoglossicida* 2,4-D (2,4-D), humic substances (HSs) and their combinations (2,4-D + HSs).

Groups	Groups	{1}	{2}	{3}	{4}	{5}
	Mean Values	0.31710	0.28940	0.35550	0.35210	0.44030
1	control		0.476096	0.341457	0.367674	0.002716 **
2	WD control	0.476096		0.114669	0.127817	0.000307 **
3	WD, 2,4-D	0.341457	0.114669		0.925533	0.028625 *
4	WD, HSs	0.367674	0.127817	0.925533		0.027200 *
5	WD, 2,4-D + HSs	0.002716 **	0.000307 **	0.028625 *	0.027200 *	

**Table 8:** ANOVA test for shoot ABA content followed by Duncan test. Values in the cells correspond to p for the difference between the mean values; *p* ≤ 0.05 is marked with *; *p* ≤ 0.01—with ** for control plants untreated with either bacteria or HSs and plants grown under water deficit (WD): control, plants treated with *Pseudomonas plecoglossicida* 2,4-D (2,4-D), humic substances (HSs) and their combinations (2,4-D + HSs).

Groups	Groups	{1}	{2}	{3}	{4}	{5}
	Mean Values	51.934	60.684	42.119	76.802	44.652
1	control		0.282048	0.473674	0.087514	0.570701
2	WD control	0.282048		0.091436	0.460178	0.121308
3	WD, 2,4-D	0.473674	0.091436		0.020632 *	0.843386
4	WD, HSs	0.087514	0.460178	0.020632 *		0.029786 *
5	WD, 2,4-D + HSs	0.570701	0.121308	0.843386	0.029786 *	

**Table 9:** ANOVA test for root ABA content followed by Duncan test. Values in the cells correspond to p for the difference between the mean values; *p* ≤ 0.05 is marked with *; *p* ≤ 0.01—with ** for control plants untreated with either bacteria or HSs and plants grown under water deficit (WD): control, plants treated with *Pseudomonas plecoglossicida* 2,4-D (2,4-D), humic substances (HSs) and their combinations (2,4-D + HSs).

Groups	Groups	{1}	{2}	{3}	{4}	{5}
	Mean Values	36.690	67.053	33.294	66.890	30.302
1	control		0.005936 **	0.757047	0.005054 **	0.569397
2	WD control	0.005936 **		0.003481 **	0.986915	0.001657 **
3	WD, 2,4-D	0.757047	0.003481 **		0.003282 **	0.762073
4	WD, HSs	0.005054 **	0.986915	0.003282 **		0.001580 **
5	WD, 2,4-D + HSs	0.569397	0.001657 **	0.762073	0.001580**	

**Table 10:** ANOVA test for shoot cytokinins content followed by Duncan test. Values in the cells correspond to p for the difference between the mean values; *p* ≤ 0.05 is marked with *; *p* ≤ 0.01—with ** for control plants untreated with either bacteria or HSs and plants grown under water deficit (WD): control, plants treated with *Pseudomonas plecoglossicida* 2,4-D (2,4-D), humic substances (HSs) and their combinations (2,4-D + HSs).

Groups	Groups	{1}	{2}	{3}	{4}	{5}
	Mean Values	74.636	89.256	137.01	327.69	97.597
1	control		0.730084	0.116840	0.000028 **	0.600458
2	WD control	0.730084		0.192193	0.000040 **	0.834226
3	WD, 2,4-D	0.116840	0.192193		0.000274 **	0.247810
4	WD, HSs	0.000028 **	0.000040 **	0.000274 **		0.000065 **
5	WD, 2,4-D + HSs	0.600458	0.834226	0.247810	0.000065 **	

**Table 11:** ANOVA test for root cytokinins content followed by Duncan test. Values in the cells correspond to p for the difference between the mean values; *p* ≤ 0.05 is marked with *; *p* ≤ 0.01—with ** for control plants untreated with either bacteria or HSs and plants grown under water deficit (WD): control, plants treated with *Pseudomonas plecoglossicida* 2,4-D (2,4-D), humic substances (HSs) and their combinations (2,4-D + HSs).

Groups	Groups	{1}	{2}	{3}	{4}	{5}
	Mean Values	49.132	79.632	72.002	112.35	48.713
1	control		0.000049 **	0.000540**	0.000028 **	0.938284
2	WD control	0.000049 **		0.167135	0.000152 **	0.000043 **
3	WD, 2,4-D	0.000540 **	0.167135		0.000066 **	0.000518 **
4	WD, HSs	0.000028 **	0.000152 **	0.000066 **		0.000025 **
5	WD, 2,4-D + HSs	0.938284	0.000043 **	0.000518 **	0.000025 **	

## Data Availability

The data presented in this study are contained within this article.
